# Ameliorating potency of *Chenopodium album* Linn. and vitamin C against mercuric chloride-induced oxidative stress in testes of Sprague Dawley rats

**DOI:** 10.1186/s12199-019-0820-x

**Published:** 2019-11-23

**Authors:** Sarwat Jahan, Tayyaba Azad, Amina Ayub, Asad Ullah, Tayyaba Afsar, Ali Almajwal, Suhail Razak

**Affiliations:** 1 Department of Animal Sciences, Faculty of Biological Sciences, Quaid-i-Azam University, Islamabad, 45320 Pakistan; 2 Department of Community Health Sciences, College of Applied Medical Sciences, King Saud University, Riyadh, Kingdom of Saudi Arabia

**Keywords:** Vitamin C, Mercuric chloride, Oxidative stress, *Chenopodium album* Linn, Comet assay, Testes

## Abstract

**Background:**

Mercury has been documented as an industrial risk that posed a serious danger to human health. Mercury exposure results in oxidative stress that may lead to the pathogenesis of male reproductive dysfunction. The present study investigated the ameliorating potential of *Chenopodium album* L. and vitamin C against mercuric chloride-induced oxidative deterioration of reproductive functions in adult male rats.

**Methods:**

Group 1 (control) received saline. Group 2 received Mercury (0.15 mg/kg b.w, i.p) dissolved in distilled water. Groups 3 and 4 were given oral gavage of vitamin C (200 mg/kg b.w) and the ethanolic extract of *C. album* (200 mg/kg b.w) respectively, along with Mercury (0.15 mg/kg b.w, i.p). Group 5 was treated only with *C. album* (200 mg/kg b.w). After 30 days of the treatment, the rats were dissected and their testicular tissue and the cauda epididymis were used for biochemical analysis while blood plasma was used for protein determination.

**Results:**

The applied dose-treatment of Mercury-induced oxidative stress in the testis and cauda epididymis tissues of the rats was apparent by a noteworthy decrease in total protein, CAT, SOD, POD, and GST values while there was increase in ROS and TBARS levels. Furthermore, Mercury decreases daily sperm production and enhanced sperm DNA damage as noticeable by an increase in the head and tail length of comets and decrease in intact DNA. There was no significant effect on the body weight and the weight of the reproductive tissues. Treatment with *C. album* significantly ameliorated the total protein, ROS, and TBARS content. Similarly, the level of CAT, SOD, POD, and GST was significantly improved and the daily sperm production was significantly increased. Furthermore, *C. album* administration significantly protected Mercury-induced sperm DNA damage. The results of the extract treatment group were compared with those of vitamin C in detoxifying the oxidative stress and restoring the sperm parameters.

**Conclusion:**

*C. album* showed protection against Mercury-induced oxidative stress by ameliorating antioxidant enzyme activity, daily sperm production, and DNA damage in rat testes. This suggests that *C. album* could be beneficial against toxicity induced by an environmental toxicant.

## Background

Mercury has been utilized by humans since long and is still considered as one of the most important metalloids. Mercury is present naturally in the environment in three forms: elemental, inorganic as well as organic [1]. Mercury enters into the atmosphere via two major sources: natural and anthropogenic. One of the most important natural sources of mercury is volcanoes, while other natural processes include outgassing of soil and water bodies, biomass burning, and geothermal processes [2]. Mercuric chloride and methyl mercury have been declared to be highly carcinogenic by EPA [3]. Approximately 2200 metric tons of mercury have been assessed to be released into the atmosphere annually [4]. Elevated levels of mercury can impair the brain, nerves, kidneys, muscles of adults, and the developing fetus [5–7]. Bronchitis, asthma, and temporary respiratory problems have been occurred due to inhalation or exposure to mercury vapors [8]. Mercury exposure intoxicated reproductive activity via altering the hypothalamus-pituitary-adrenal and gonadal axis via impairing the circulating levels of follicle-stimulating hormone (FSH), luteinizing hormone (LH), inhibin, estrogen, progesterone, and the androgens [8, 9]. Furthermore, occupational exposure to mercury vapors leads to infertility in both men and women and erectile dysfunction [10]. The mercury-mediated pathogenesis of reproductive functions might be attributed to the abridged antioxidant defense system [11]. Hence, treatment with antioxidants might ameliorate or reduce the adverse effects of mercury on reproductive ailments.

*Chenopodium album* Linn. (Family *Chenopodiaceae*) *Bathua sag* is a native plant of western Asia. *C. album* belongs to genus Chenopodium, is a globally distributed plant, and has about 250 species. In India, it is cultivated as a vegetable. *C. album* grows naturally as a weed in the fields of wheat, barley, mustard, gram, and other crops. *C. album* is conventionally used as anthelmintic, cardiotonic, carminative, digestive, diuretic, and laxative [12–14]. It is widely used in the treatment of peptic ulcer, dyspepsia, flatulence, strangury, pharyngopathy, splenopathy, ophthalmopathy, and general debility. Traditionally, *C. album* is used for the cure of hepatic disorders, spleen enlargement, intestinal ulcers, and burns have also been recognized [15].

*C. album* is a rich source of nutrients, antioxidants, and essential dietary elements [11, 16]. Vitamin C and β-carotene are detected in the young shoots and mature plants of *C. album* [17]. Leaves contain trypsin inhibitor activity, phenols, tannins, saponin, phytic acid, phytate phosphorus, alkaloids, flavonoids, oxalates, oil, and proteins [18]. The plant is a source of energy, proteins, carbohydrates, ascorbic acid and beta carotene, and minerals, such as potassium, sodium, calcium, phosphorous, magnesium, iron, and zinc. It has been screened for nutritional, phytochemical, antioxidant, and antibacterial activity [19]. *C. album* possesses aphrodisiac activity, cures male infertility, and increases the copulatory efficiency respectively [20]. Male rats and mice treated with ethanolic seed extract of the plant exhibited increased reproductive organs and body weight gain parallel with increased sperm count and sexual performance. There was an improvement in sexual behavior and performance and an increase in sperm count in male mice and rats [20]. Nevertheless, *C. album* seed extract (CAE) also caused sperm death due to oxidative damage induced by the generation of ROS [21]. The DNA protective ability has also been evaluated in an animal model [22].

Vitamin C is known for its antioxidant and DNA protecting properties. Vitamin C is an excellent radical scavenger that may neutralize the possible spermicidal and genotoxic effects of various free radicals and protect against the radicals, which are toxic by-products of many metabolic processes [23]. Vitamin C reduces sperm agglutination and increases the fertility of men, while improving the sperm quality and male fertility [23]. Studies have revealed the protective benefits of vitamin C against heavy metals [24].

## Methods

### Plant extract preparation

*Chenopodium album* Linn was collected from the agricultural and cultivated fields of Charsadda, Pakistan. The plant was identified by Dr. Mir Ajab (Department of Plant Sciences, Quaid-i-Azam University Islamabad) and the voucher specimen (0623451) was deposited at the Herbarium of Pakistan, Quaid-i-Azam University Islamabad. Seeds, together with the funicles, were air dried under the shade. They were stored in sealed bags until the preparation of the extract. Seeds were incubated at 25 °C, ground, and then sieved. The dried powder was then added to ethanol and placed at 4 °C in the dark for a week and was stirred every day for 30 min. The mixture was filtered with Whatman filter paper number 41 having a diameter of 150 mm. The filtrate was placed at room temperature until the solvent was evaporated. A 200 mg/kg dose of *C. album* was selected based on the previous study in which the effect of the same dose was observed on sexual behavior and sperm count in male rats [25, 26]. The ethanolic extract was suspended in 2% polyvinyl pyrrolidone and 100% stock solution was prepared.

### Animals

A total of 35 adult male Sprague Dawley rats (*Rattus norvegicus*), allowing an average weight of 240 ± 15 g, were obtained from animal house of Quaid-i-Azam University Islamabad that were bred and kept in stainless steel cages. The animals were having free access to standard laboratory feed (Rat feed SPR 1002, Islamabad Feed Company, Pakistan) and water ad libitum*.* They were kept in an animal house according to the standard guideline, i.e., the temperature was maintained at 25 °C with light and dark cycle of 12 h. Before the onset of the study, the experimental protocol was approved by the ethical committee of the Department of animal sciences, Quaid-i-Azam University, Islamabad.

### Experimental work

Animals were divided into five groups (*n* = 7). The first group received saline through a gavage (control group). The second group was treated with an i.p injection of Mercury (0.15 mg/kg b.w) dissolved in distilled water (Mercury group). The third group was the positive control group that received an i.p injection of Mercury (0.15 mg/kg b.w) dissolved in distilled water and vitamin C (200 mg/kg b.w) orally (vitamin C group). The fourth group was the co-treatment group that was treated with an i.p injection of Mercury (0.15 mg/kg b.w) dissolved in distilled water and *Chenopodium album* L. extract (200 mg/kg b.w) through gavage (extract treatment group). The fifth group received only *Chenopodium album* L. extract (200 mg/kg b.w) through gavage (extract group).

### Chemical and applied dose-treatment

Mercuric chloride (Mercury) was purchased from BDH Chemicals (Ltd Pool, England). The applied dose-treatment (0.15 mg/kg) of mercuric chloride (Mercury) was carried out according to Schionning and Larsen [27]. Vitamin C was bought from VWR Prolab (Geldena, Kseban), and 200 mg/kg dose was selected according to Uzun et al. [28], showed same dose was effective against testicular toxicity induced by malathion in male rats. All the animals received doses for 30 days. After 24 h of the last dose administration, animals were euthanized by cervical dislocation.

### Sample preparation

The blood was collected directly from the heart of each animal in heparinized syringes. Plasma was obtained by centrifugation of the blood at 3000 rpm for 10 min and was kept at – 20 °C until hormonal analysis. Testes, epididymis, and prostate were weighed. The relative organ weight was calculated using the following formula:

$$ \mathrm{Relative}\ \mathrm{organ}\ \mathrm{weight}=\mathrm{Organ}\ \mathrm{weight}/\mathrm{body}\ \mathrm{weight} $$

The left testis and epididymis were kept at − 70 °C for comet assay. The right testis and epididymis were utilized for histology. Both testis and epididymis were fixed 10% formalin and processed for histological investigation. Extra fresh-testicular tissue (180 mg) was homogenized in 10 volumes of 100 mM KH_2_PO_4_ buffer containing 1 mM EDTA, pH 7.4 and centrifuged at 12,000×*g* for 30 min at 4 °C. The supernatant was collected and used for the following assays.

### Protein estimation

The total protein content of testicular tissue was determined by total protein kit (AMP diagnostics) using bovine serum albumin as a standard.

### Hormone analysis

The plasma testosterone level was determined by ELISA kits (AmgenixInc, USA) according to the guideline provided by the manufacturer.

### Biochemical analysis

Plasma urea nitrogen level (BUN), creatinine, cholesterol, triglyceride, high-density lipoprotein, and low-density lipoprotein concentrations were determined by using the AMP diagnostic kit (AMEDA labordiagnostikGmbh, Austria), and were analyzed on Picco 5 chemistry analyzer.

### Assessment of oxidative stress markers

Reactive oxygen species (ROS) activity was detected in testicular tissue of control and treated animals. Absorbance was checked at 505-nm Smart Spec TM plus Spectrophotometer. The amount of TBARS as an index of lipid peroxidation was assessed by measuring the optical density of the supernatant at 535 nm using a spectrophotometer against a reagent blank using the method of Tyan and coworkers [29]. The results were expressed as nM TBARS/min/mg tissue at 37 °C using a molar extinction coefficient of 1.56 × 10^5^/Mcm.

### Assessment of antioxidant status

Catalase activity was determined by the method of Afsar and colleagues [30]. Changes in absorbance of the reaction mixture at 240 nm were determined after 1 min. One unit of CAT activity was defined as an absorbance change of 0.01 as units/min. Peroxidase activity was determined by the method described previously [31]. Changes in absorbance of the reaction solution at 470 nm were determined. One unit of POD activity was defined as an absorbance change of 0.01 as units/min. Superoxide dismutase activity was estimated following previous protocols [30]. Amount of chromogen formed was measured by recording the color intensity at 560 nm. Results are expressed in units/mg protein. For glutathione-S-transferase activity, changes in the absorbance of the reaction mixture were recorded at 340 nm and enzyme activity was calculated as nM CDNB conjugate formed/min/mg protein using a molar extinction coefficient of 9.6 × 103 M^−1^ cm^−1^.

### Assessment of daily sperm production

Spermatids were counted by the method described previously [32]. Shortly, frozen testis was thawed at room temperature, tunica albuginea was removed, and then parenchyma was weighed. It was homogenized in 5 ml of 0.9% NaCl solution (which included 0.5% triton X-100). Twenty microliters of the sample was placed into Neubauer chambers and quantity of late spermatids was counted under the microscope at × 400 magnification. This provides the total number of spermatids per testis. The number was used for determination of the number of spermatids per each gram (g) of testicular tissue which is the efficacy of sperm production. For the calculation of daily sperm production (DSP), the number of spermatids (per testis and per g of testis) was divided by 6.3 (DSP = Y/6.3).

### DNA damage analysis

#### Comet assay

DNA damage was estimated by comet assay and separate spermatozoa were evaluated by using a modified basic Single Cell Gel Electrophoresis (SCGE/comet assay) according to the method of [33]. The basic steps of comet assay include lysis of membranes to release DNA, single-strand DNA formed by contact to alkali (pH − 13), neutral electrophoresis, DNA staining and analysis, and comet scoring.

### Histopathological examination

Histology of testis and epididymis was performed after removal of tissues from animals and the following steps were carried. Tissues were placed in 10% formalin for 24–48 h and then dehydrated in the ascending grades of ethyl alcohol, cleared in xylene, and mounted in molten paraffin wax 58–62 °C. Seven-micrometer histological sections were cut and stained with hematoxylin and eosin and observed under the light microscope (Nikon, 187842, Japan) at × 40 and microphotography was performed by Leica LB microscope connected to Olympus Optical Co. LTD, Japan.

### Morphometric analysis

For morphometric studies, the seminiferous tubule diameter and germ cell height were measured by using Image J software (National Institute of Health, Bethesda, MD, USA) [34]. Images were taken at × 20 and × 40 and morphometry was done using software Image J. Area of the seminiferous tubule, epididymis tubules, and interstitial space was determined by planimetry, using Image J software. An area in μm^2^ was calculated by the method of Islam et al., (2010) [34] and according to the [35]. Shortly 25 pictures at × 20 per animal of the known area were selected and the area of seminiferous tubules, epididymis tubule, and interstitial space was determined by free selection tool of the software. The area % age was calculated by the formula:

% *As* = *As* × 100/*T*

where *As* is area covered by seminiferous tubules. *T* is the total area of the field. Percentage of the mean area was analyzed for comparison between treated and control groups and was reported.

### Statistical analysis

Data were subjected to one-way analysis of variance (ANOVA) followed by Tukey’s test for comparison of different groups using GraphPad Prism software. All the results are shown as Mean ± SEM and the significance level was set at < 0.05.

The sample size for the current study was calculated by the resource equation method [36] by using the following formula:

*E* = Total number of animals − Total number of groups

where *E* is the degree of freedom of analysis of variance (ANOVA). The value of *E* should lie between 10 and 20 to increase the chance of getting a more significant result. As this method is based on ANOVA, it is applicable to all animal experiments [37].

In the present study, we made four groups with seven animals each.

$$ E=\left(7\times 5\right)-5\;E=\left(7\times 5\right)-5 $$

$$ E=35-5=30\kern0.2em E=35-5=30 $$

This sample size is adequate as chances of death of animals cannot be ignored.

## Results

### Body and organ weights

Mercury treatment significantly reduced body weight (185 ± 6.23) compared with the normal control group (248.4 ± 4.60). The plant alone group showed non-significant different in body and organ weight compared with the control group. There was a significant weight gain in the animals of the vitamin C and the extract groups (*p* < 0.0001) compared with the Mercury group but there was a non-significant change when compared with each other (Table 1). All experimental animals showed no significant change in reproductive organ weight.

**Table 1:** Effect of different treatments on body and reproductive organ weights

Treatments	Initial body weight (g)	Final body weight (g)	Relative organ weights				
			Left testis weight (mg)	Right testis weight (mg)	Left epididymis weight (mg)	Right epididymis weight (mg)	Prostate weight (mg)
Control	225.20 ± 5.54	248.4 ± 4.60	5.03 ± 0.122	4.83 ± 0.10	1.85 ± 0.077	2.01 ± 0.081	1.97 ± 0.274
Mercury	231.60 ± 12.70	185 ± 6.23***	5.83 ± 0.134	5.35 ± 0.121	2.92 ± 0.091	2.65 ± 0.087	3.95 ± 0.243
Mercury + vit C	226.20 ± 5.70	247 ± 5.36^+++^	4.91 ± 0.125	5.02 ± 0.131	2.05 ± 0.08	2.11 ± 0.091	3.16 ± 0.22
Mercury + *C. album*	230.80 ± 8.45	250.2 ± 4.62^+++^	4.84 ± 0.121	4.96 ± 0.132	1.68 ± 0.071	1.92 ± 0.086	4.20 ± 0.31
*C. album* alone	227.5 ± 6.45	249 ± 4.56^+++^	5.34 ± 0.138	5.10 ± 0.134	1.94 ± 0.09	2.14 ± 0.077	2.08 ± 0.25

Values are expressed as mean ± SEM; “***” indicating significance level from control group at *p* < 0.001 probability levels. “^+++^” indicates significance level from Mercury group at *p* < 0.001 probability level

### Biochemical analysis of plasma

In Table 2, the summarized changes in plasma levels of urea nitrogen (BUN), creatinine, cholesterol, triglyceride, high-density lipoprotein (HDL), and low-density lipoproteins (LDL) were estimated in the experimental group. No significant difference observed between the values of serum creatinine among all compared groups. A significant rise in BUN and cholesterol (*p* < 0.01) was observed in Mercury-intoxicated group as compared with the control group. However, Mercury + *C. album* and Mercury + vitamin C treatments corrected BUN and cholesterol levels as compared with Mercury alone-treated group (*p* < 0.001 and *p* < 0.05 respectively).

**Table 2:** Protective effect of *C. album* extract against HgCl_2_-induced alteration in plasma BUN, creatinine, cholesterol, triglyceride, HDL, and LDL levels

Parameters	Control	Mercury	Mercury + vit C	Mercury + *C. album*	*C. album* alone
BUN (mg/dl)	24.54 ± 1.44	34.03 ± 1.90**	30.49 ± 1.64	23.95 ± 1.20^+++, −^	23.74 ± 1.50^+++^
Creatinine (mg/dl)	2.13 ± 0.70	5.13 ± 0.97	4.97 ± 0.76	3.41 ± 0.84	2.11 ± 0.73
Cholesterol (mg/dl)	119.48 ± 1.93	140.78 ± 2.32***	157.13 ± 2.14***, ^+++^	196.17 ± 2.54***,^+++^,^−−−^	116.28 ± 1.76^+++^
Triglyceride (mg/dl)	27.88 ± 1.86	34.42 ± 1.50*	29.94 ± 1.69	41.21 ± 1.31***, ^+^, ^−−−^	26.81 ± 1.79^+^
HDL (mg/dl)	138.24 ± 1.60	95.40 ± 1.27***	134.15 ± 1.93***, ^+++^	130.62 ± 1.97***, ^+++^	140.04 ± 1.59^+++^
LDL (mg/dl)	4.32 ± 0.04	18.50 ± 1.54	9.4 ± 2.81**, ^++^	8.31 ± 1.51**, ^++^	4.12 ± 0.06^++^
Testosterone (ng/ml)	5.74 ± 0.19	4.51 ± 0.15***	6.73 ± 0.96^+++^	8.08 ± 0.89^+++^	5.77 ± 0.21^+++^

Values are expressed as mean ± SEM; “*,” “**,” and “***” indicating significance level from control group at *p* < 0.05, *p* < 0.01, and *p* < 0.001 probability levels. “^+^,” “^++^,” and “^+++^” indicate significance level from Mercury group at *p* < 0.05, *p* < 0.01, and *p* < 0.001 probability levels, whereas “^−^,” “^−−^,” and “^−−−^” indicate significance from Mercury + vit C group at *p* < 0.05, *p* < 0.01, and *p* < 0.001 probability levels, respectively

A significant increase was observed in the triglyceride values (*p* < 0.05) when the Mercury group was compared with the control group. However, a significant difference (*p* < 0.001) was observed in the Mercury + *C. album*-treated group as compared with control. In the HDL values, there was a significant increase (*p* < 0.001) observed in the Mercury + *C. album* and Mercury + vitamin C groups; however, the values of HDL decreased in the Mercury alone group in comparison with the control. On the other hand, there was a significant decrease (*p* < 0.01) observed in the values of LDL in the Mercury + *C. album* and Mercury + vitamin C groups; however, the values of LDL increased in the Mercury alone group in comparison with the control. The plant alone group showed similarity in abovementioned parameters as the control group.

### Effect on testosterone concentration

**Fig. 1:**
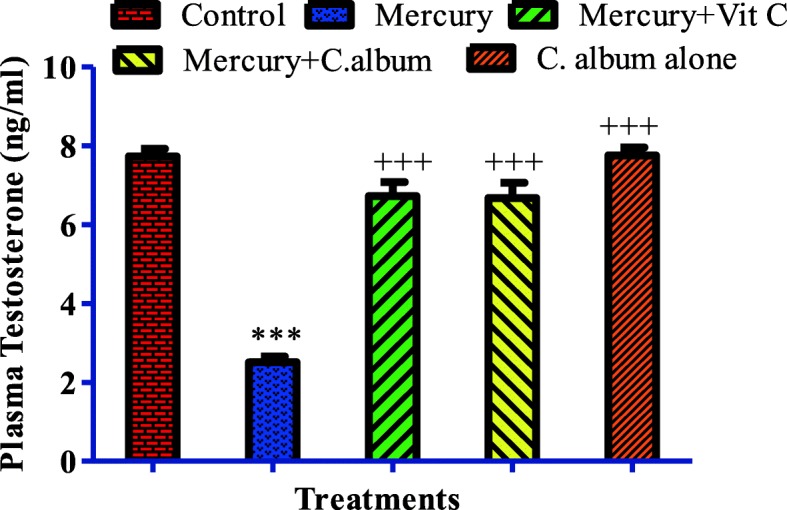
Effect of different treatments on plasma testosterone concentration

The changes in blood plasma testosterone concentration in male Sprague Dawley rats are given in Fig. 1. The Mercury-treated group showed significant decrease (*p* < 0.001) in plasma testosterone levels in comparison with the control group. Significant increase (*p* < 0.001) in testosterone levels was observed in Mercury + *C. album* and Mercury + vitamin C-treated groups as compared with Mercury-intoxicated group.

### Effect on protein concentration

Protein concentration in various experimental groups is shown in Table 3. A significant decrease (*p* < 0.001) in total protein values of Mercury, vitamin C, and extract group (*p* < 0.01) was observed when compared with the control. Total protein values were significantly higher (*p* < 0.01) in vitamin C and the extract group compared with the Mercury-treated group. There was no significant change in protein values between vitamin C and the extract group.

**Table 3:** Protective effect of *C. album* extract against HgCl_2_-induced alteration in tissue protein content, oxidative stress markers, and antioxidant status

Parameters	Control	Mercury	Mercury + vit C	Mercury + *C. album*	*C. album* alone
Total protein (mg/g of tissue)	72.41 ± 2.26	43.65 ± 1.76a***	57.46 ± 2.68a**b**	59.30 ± 2.11a**b**	73.01 ± 2.16^+++^
ROS (U/min)	1.26 ± 0.28	3.15 ± 0.30a***	1.71 ± 0.14b**	2.07 ± 0.09b*	1.23 ± 0.26^++++^
TBARS (nmol/min/mg)	3.12 ± 0.89	9.55 ± 0.63a***	2.32 ± 0.32b***d**	5.72 ± 0.49a*b**	3.02 ± 0.81^+++^
CAT (U/min)	35.74 ± 3.33	22.87 ± 1.65a**	31.36 ± 2.29	29.07 ± 1.36	36.79 ± 3.13^++^
SOD (U/min)	19.77 ± 1.11	9.16 ± 1.00a**	14.45 ± 1.40	15.82 ± 2.57b*	20.71 ± 1.20^++^
POD (nmol)	16.05 ± 0.95	3.78 ± 0.47a***	13.07 ± 1.14b***	11.55 ± 0.95a*b***	17.15 ± 0.91^+++^
GST (U/mol/mg)	42.21 ± 1.95	22.74 ± 1.27a**	41.19 ± 3.68b**	29.85 ± 4.03a*	43.29 ± 1.89^++^

Values are expressed as mean ± SEM; “*,” “**,” and “***” indicating significance level from control group at *p* < 0.05, *p* < 0.01, and *p* < 0.001 probability levels. “^+^,” “^++^,” and “^+++^” indicate significance level from Mercury group at *p* < 0.05, *p* < 0.01, and *p* < 0.001 probability levels, whereas “^−^,” “^−−^,” and “^−−−^” indicate significance from Mercury + vit C group at *p* < 0.05, *p* < 0.01, and *p* < 0.001 probability levels, respectively

### Effect on oxidative stress markers and antioxidant status

The value of ROS and TBARS in the Mercury-treated group increased significantly (*p* < 0.05) as compared with that of the control group (Table 3). The values were significantly reduced in vitamin C and Mercury + *C. album* as compared with that of the Mercury group. No significant change in the ROS values of vitamin C and Mercury + *C. album* was observed when compared with the control group and also with each other. However, the TBARS values were significantly reduced (*p* < 0.01) in the vitamin C group in comparison with the Mercury + *C. album*. For CAT and SOD, values were significantly reduced (*p* < 0.01) as compared with the control. But for both values, vitamin C group showed no significant change either with the control or Mercury-treated group. Also the Mercury + *C. album* showed the non-significant result for CAT but significant (*p* < 0.05) for SOD values when compared with Mercury group. For CAT and SOD, vitamin C group and the Mercury + *C. album* groups did not show any significant alterations when compared with each other. POD and GST values also showed a significant decrease (*p* < 0.001 and *p* < 0.01 respectively) in values for the Mercury group as compared with the control. The increase in POD values was significant (*p* < 0.001) in both vitamin C and the extract group as compared with the control group. For GST, vitamin C group showed significant (*p* < 0.01) result but not the *C. album* group when compared with Mercury-treated group. Although the *C. album* group showed significant results (*p* < 0.05) for both POD and GST values when compared with the control but no significant result was obtained when vitamin C and the *C. album* groups were compared with each other. The plant alone group showed similar results as control group.

### Daily sperm production and efficiency

Mercury group showed a significant decrease (*p* < 0.001) in DSP and efficiency of DSP as compared with the control group (Table 4). Vitamin C and the *C. album* group showed significantly increased values (*p* < 0.001) for both parameters when compared with the Mercury group. For efficiency of DSP, vitamin C showed significant results (*p* < 0.05) as compared with the control.

**Table 4:** Protective effect of *C. album* extract against HgCl_2_-induced alterations in DSP and DNA damage

Parameters	Control	Mercury	Mercury + vit C	Mercury + *C. album*	*C. album* alone
DSP parameters					
DSP/100 mg testis (10^5^)	17.05 ± 1.14	6.20 ± 1.07a***	17.60 ± 0.71b***	15.40 ± 0.58b***	17.67 ± 1.19^+++^
DSP efficiency (10^5^)	225 ± 7.88	120 ± 4.93a***	256.60 ± 7.45a*b***	254.60 ± 8.09a*b***	230 ± 6.99^++++^
Comet assay parameters					
Comet head length (μm)	91.40 ± 9.09	149.40 ± 7.38a***	125 ± 8.62a*	119.20 ± 7.32	92.01 ± 8.49^+++^
Comet tail length (μm)	16.80 ± 2.16	30.20 ± 1.47a*	26.20 ± 3.46	22.80 ± 3.41	16.20 ± 2.36^+^
%DNA in head	91.51 ± 0.83	89.47 ± 1.87	91.04 ± 2.01	94.58 ± 1.26	92.11 ± 0.76
%DNA in tail	5.32 ± 0.37	8.01 ± 0.33a*	8.17 ± 0.95a*	9.56 ± 0.24a***	5.30 ± 0.34+
Tail movement	1.05 ± 0.30	2.31 ± 0.31	1.95 ± 0.36	1.09 ± 0.39	1.01 ± 0.27

Values are expressed as mean ± SEM; “*,” “**,” and “***” indicating significance level from control group at *p* < 0.05, *p* < 0.01, and *p* < 0.001 probability levels. “^+^,” “^++^,” and “^+++^” indicate significance level from Mercury group at *p* < 0.05, *p* < 0.01, and *p* < 0.001 probability levels, whereas “^−^,” “^−−^,” and “^−−−^” indicate significance from Mercury + vit C group at *p* < 0.05, *p* < 0.01, and *p* < 0.001 probability levels, respectively

### DNA damage analysis by comet assay

**Fig. 2:**
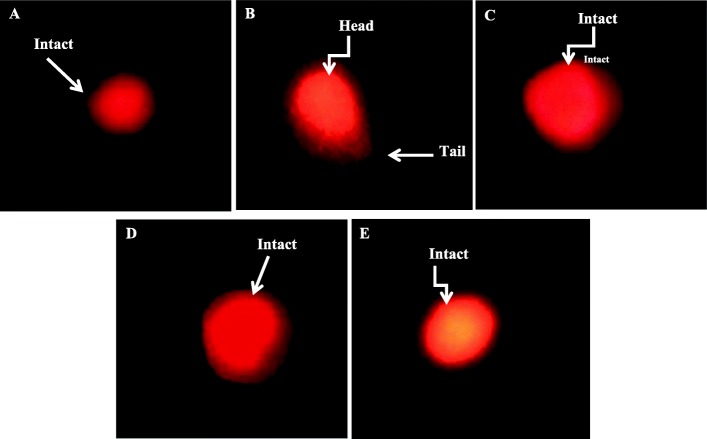
Photomicrograph (× 40) of sperm DNA damage determined by comet assay in different treatment groups after 30 days of treatment showing control group with more intact DNA (**a**), Mercury group having comets with short tails (**b**), vitamin C-treated group (**c**), and extract-treated group (**d**) representing somewhat intact DNA compared with the treated group

Head and the tail length was significantly (*p* < 0.001 and *p* < 0.05 respectively) increased in Mercury-treated group compared with the control (Table 4; Fig. 2). Vitamin C showed a significant result for head length (*p* < 0.05) but the non-significant result for tail length when compared with the control. The *C. album* group showed non-significant results for both head and tail length either compared with the control or Mercury group. %DNA in the head showed no significant result for any group. %DNA in the tail showed a significant increase (*p* < 0.05 and *p* < 0.001) in all the treated groups as compared with the control. Results were non-significant when vitamin C and the extract groups were compared with Mercury-treated group. The tail movement also showed no significant result for any treated group. Also, no significant result was obtained for any parameter when vitamin C and the *C. album* groups were compared with each other.

### Effect on tissue morphology

**Fig. 3:**
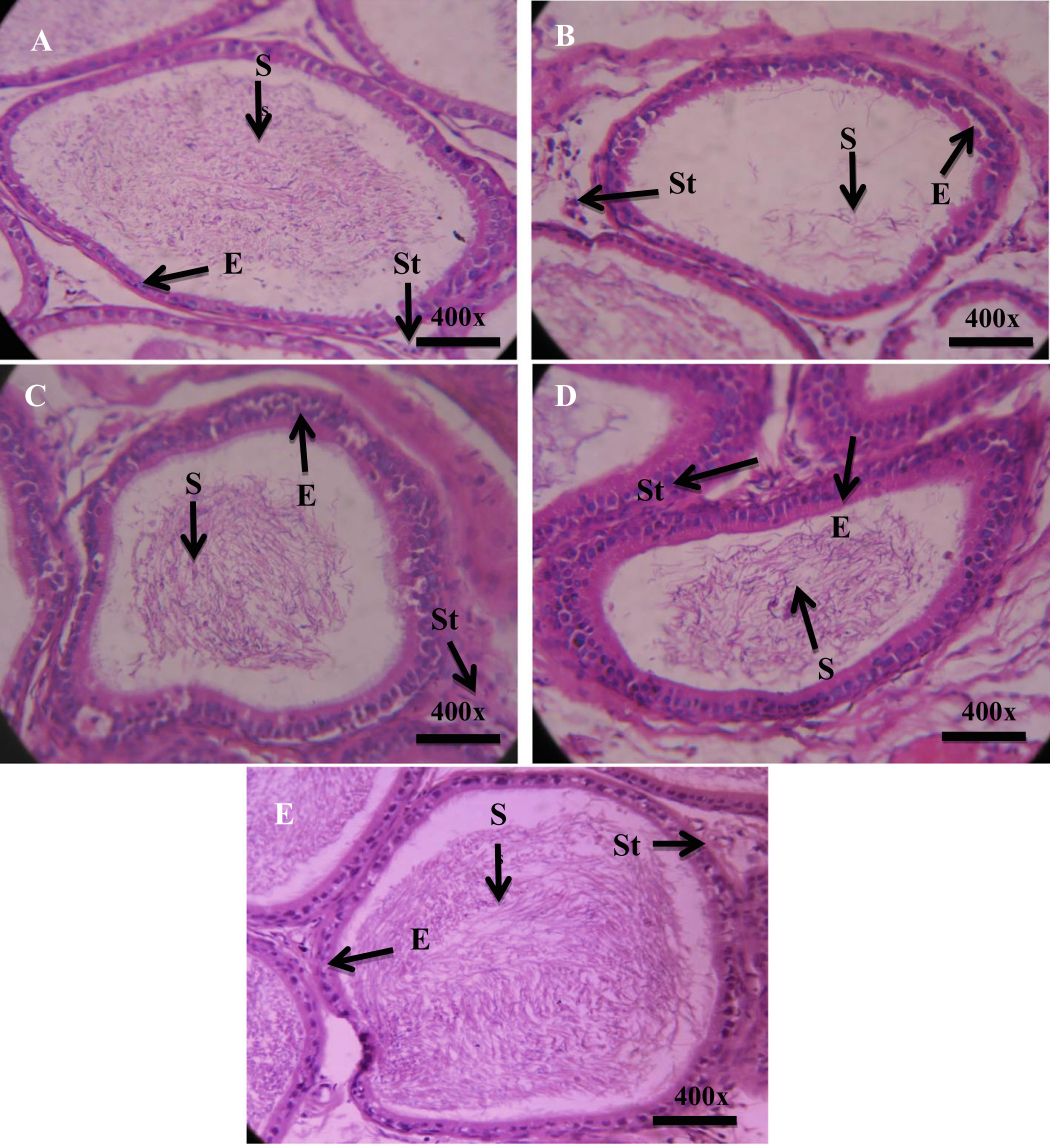
Photomicrograph of seminiferous tubules of adult male rat testes. Control group showing compact tubules, spermatid inside lumen, normal germ cell proliferation in epithelium (**a**), Mercury group shows tubules with empty lumen degenerated epithelial layer and increased interstitial spaces (**b**). Vitamin C-treated showing less damage to epithelium lumen filled with spermatid and interstitial spaces (**c**). *C. album* co-treated shows minimal damage to epithelium and tubules, with less interstitial spaces (**d**). Magnification × 40. SP, spermatogonia; ES, elongated spermatids; IS, interstitial space; E, epithelium; × 40 by × 10 = × 400

The histological examination exhibited normal spermatogenesis and closely arranged seminiferous tubules in the control and plant alone groups (Fig. 3). Proliferating germ cells showing different stages of spermatogenesis were surrounded by thick stratified germinal epithelium. The tubular lumen was narrow containing mature spermatozoa. Oval-, round-, or irregular-shaped Leydig cells and large blood vessels were present in the interstitial spaces surrounding the seminiferous tubules (Fig. 3). The Mercury-exposed group showed degradation in seminiferous tubular diameter showing deteriorated spermatogenesis as compared with control, Mercury + vitamin C, and Mercury + *C. album* co-treatment groups. An increment in interstitial spaces with wide lumen and increase in germinal epithelial spaces are evident due to lack of germ cells along epithelium as shown in Fig. 2. The Mercury + vitamin C and Mercury + *C. album* experimental groups exhibited obvious alteration than the Mercury-treated group (Fig. 3). Restoration of normal spermatogenesis was obvious in most seminiferous tubules, and round and elongated spermatids were observed. Reduction in the interstitial spaces was caused by an increase in Leydig cell number. The release of mature spermatids into lumen caused the reduction in lumen diameter (Fig. 3).

No significant changes were observed in seminiferous tubular diameter among the control and treated groups. A highly significant decrease in the percentage area of seminiferous tubules (*p* < 0.001) was observed in the Mercury group when compared with the control group. Similarly, a highly significant increase (*p* < 0.001) in an area of seminiferous tubules was observed in Mercury + vitamin C and Mercury + *C. album* when compared with Mercury alone group. Percentage of the areas of lumen showed the highly significant increase (*p* < 0.001) in Mercury-treated group when compared with the control group (Table 5). A statistically significant decrease in lumen area (*p* < 0.001) was observed in Mercury + vitamin C and Mercury + *C. album* when compared with Mercury alone group. Percentages of the area of interstitium have shown a highly significant increase in the interstitium of (*p* < 0.001) in Mercury alone group as compared with control (Fig. 3). The vitamin C and *C. album* groups showed a highly significant decrease in the area (*p* < 0.001) when compared with Mercury alone group. However, Mercury-treated group showed a relatively decreased value of epithelium. The mean diameter of caput is given in showing a significant increase of diameter (*p* < 0.001) in Mercury-treated group when compared with control group, whereas Mercury + vitamin C also showed a highly significant increase (*p* < 0.01) when compared with control. No significance was observed in percentage areas of lumen, interstitium, and epithelium.

**Table 5:** Effect of *C. album* extract and vitamin C treatment on testicular morphometric parameters in different experimental groups

Parameters	Control	Mercury	Mercury + vit C	Mercury + *C. album*	*C. album* alone
Area of seminiferous tubules (%)	81.50 ± 2.01	25.65 ± 2.26***	80.68 ± 3.74^+++^	78.05 ± 4.48^+++^	81.80 ± 2.15
Area of lumen (%)	22.88 ± 1.82	57.03 ± 5.46***	14.31 ± 1.50^+++^	12.59 ± 1.80^+++^	22.04 ± 1.57
Area of interstitium (%)	18.50 ± 2.01	74.35 ± 2.26***	19.32 ± 3.74^+++^	21.95 ± 4.48^+++^	19.00 ± 2.22
Area of epithelium (%)	77.12 ± 1.82	42.97 ± 5.46	85.69 ± 1.50	87.41 ± 1.80	77.52 ± 1.91
Area of tubular diameter (μm)	253.73 ± 6.61	255.08 ± 5.26	271.00 ± 4.58	273.25 ± 8.97	260.13 ± 5.91
Area of caput tubules (%)	78.89 ± 6.03	65.18 ± 4.07	63.87 ± 4.41	76.91 ± 4.06	79.09 ± 6.13
Area of cauda tubules (%)	74.88 ± 2.73	67.24 ± 2.65	74.63 ± 3.29	84.35 ± 2.50+	75.01 ± 2.54
Area of caput lumen (%)	38.26 ± 2.73	52.07 ± 2.28	49.95 ± 10.08	43.07 ± 1.83	37.96 ± 2.13
Area of cauda lumen (%)	70.08 ± 1.75	63.12 ± 4.39	70.48 ± 1.18	69.29 ± 2.02	70.38 ± 1.78
Area of caput interstitium (%)	21.11 ± 6.03	34.82 ± 4.07	36.13 ± 4.41	28.10 ± 4.06	21.31 ± 6.23
Area of cauda interstitium (%)	25.12 ± 2.73	32.76 ± 2.65	25.37 ± 3.29	15.65 ± 2.50+	25.62 ± 2.98
Area of caput epithelium (%)	61.74 ± 2.73	47.93 ± 2.28	50.05 ± 10.08	56.93 ± 1.83	62.04 ± 2.99
Area of cauda epithelium (%)	29.92 ± 1.75	36.88 ± 4.39	29.52 ± 1.18	30.71 ± 2.02	30.32 ± 2.25
Ductular diameter of caput (μm)	170.25 ± 8.46	198.59 ± 6.38*	202.58 ± 6.24**	181.82 ± 5.17	171.05 ± 7.76
Ductular diameter of cauda (μm)	291.71 ± 7.31	254.23 ± 4.96***	274.12 ± 6.66	308.62 ± 6.77^+++ − −^	292.31 ± 7.11

Values are expressed as mean ± SEM; “*,” “**,” and “***” indicating significance level from control group at *p* < 0.05, *p* < 0.01, *p* < 0.001 probability levels. “^+^,” “^++^,” and “^+++^” indicate significance level from Mercury group at *p* < 0.05, *p* < 0.01, and *p* < 0.001 probability levels, whereas “^−^,” “^−−^,” and “^−−−^” indicate significance from Mercury + vit C group at *p* < 0.05, *p* < 0.01, and *p* < 0.001 probability levels, respectively

**Fig. 4:**
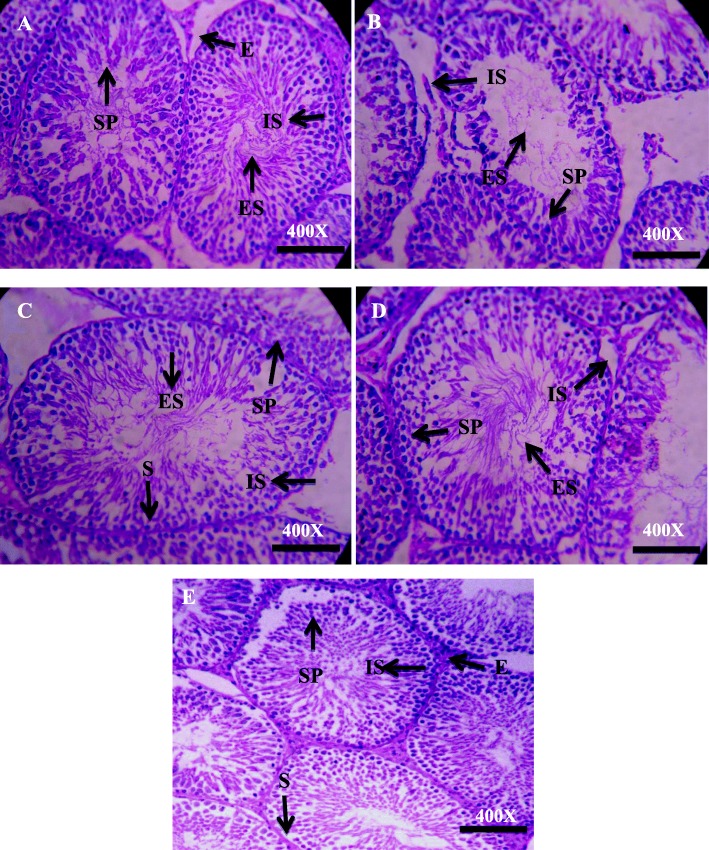
Photomicrograph of adult male cauda epididymis (H&E, × 40) from control group showing normal morphology of cauda epididymis, thin pseudostratified epithelium lined with stereocilia, and lumen filled with spermatozoa (**a**), Mercury-treated showing reduced pseudostratified epithelium with distorted spermatozoa and empty lumen in most of tubules (**b**), Mercury + vitamin C shows increased spermatozoa and pseudostratified epithelium (**c**), Mercury + *C. album* shows normal spermatozoa and epithelium (**d**). St, stroma; E, epithelium; S, spermatozoa; × 40 by × 10 = × 400

Histology of cauda epididymis (Table 5, Fig. 4) showed a significant decrease in the diameter of the ducts in the Mercury-treated group. The number of sperms in the lumen was decreased that caused constriction of the lumen in the Mercury group. The Mercury + vitamin C and Mercury + *C. album* showed changes when compared with Mercury-intoxicated group. Increase in diameter, area, and sperm number in cauda lumen was observed in Mercury + *C. album and* Mercury + vitamin C groups. The diameter of cauda epididymis showed a highly significant decrease (*p* < 0.001) in Mercury alone group when compared with the control. Mercury + *C. album* group showed a high significant increase (*p* < 0.001) of diameter when compared with control and significant increase (*p* < 0.01) as compared with Mercury + vitamin C. Mercury + *C. album-*treated group showed obvious significantly increased (*p* < 0.05) value of the area of tubules when compared with Mercury alone group. A significant decrease in the interstitium area was obvious in Mercury + *C. album-*treated group when compared with Mercury-intoxicated group.

## Discussion

Exposure of human and animals to either inorganic or organic forms of mercury results in the creation of reactive oxygen species (ROS) and oxidative stress, which cause cell death. Oxidative damage may consequence from declined clearance of ROS by scavenging mechanisms that negatively distress male reproductive systems of humans and animals [38]. The present study investigated the ameliorating ability of *C. album* Linn. and vitamin C against oxidative stress produced by Mercury in the reproductive tissues of male rats. Also, the effect of *C. album* was compared with vitamin C which served as the positive or standard control.

In previous literature, variable effects of Mercury on animal growth have been reported [7]. Our results are in agreement with the reports of Mehboob and coworkers, who reported a decrease in body weight in Mercury-intoxicated group [7, 39]. In the current investigation, we observed that *C. album* and vitamin C ameliorated mercury-induced weight loss. The loss of body weight in Mercury-treated animals is reported in other researches as well [40]. Improvement of body weight in *C. album* and vitamin C groups indicated that might preclude Mercury intoxication and our findings are in accord with the previous findings [41]. The testicular weight was slightly reduced in Mercury-treated animals, but overall, there was no significant change in all the groups. This is in accordance with the findings of Khan and colleagues [42]. Also, the testicular weights of vitamin C and *C. album* extract groups were almost similar to each other. Chatterjee and Pakrashi showed that the effect might be due to the greater availability of testosterone or similar steroidal compounds to gonads as *C. album* is also known to contain compounds of steroidal nature [43].

The Mercury-treated group showed a highly significant decrease in protein values. This result goes parallel with previous findings [44]. Several reasons can account for these low levels of protein like the interaction of Mercury with thiol groups of amino acids [45], through oxidative stress and possibly by indirectly inhibiting the protein synthesis. This inhibition might be due to disruption of the protein synthetic machinery [6, 46], inactivation of RNA polymerase I by binding to Mercury [47], or partly due to an autoregulatory reduction in tubulin synthesis [48]. The vitamin C and *C. album* groups have slightly higher values than the Mercury-treated group. Similar values have been observed by the previous study where higher protein values following *Chenopodium album* L. administration were observed [49].

In the current study, Mercury exposure is allied with increase oxidative stress biomarkers (ROS and TBARS) in the testis of rats accompanied by declined in antioxidant enzymes (SOD, CAT, and GST). Previous studies also validate Mercury-induced elevation in TBARS level in testicular tissue [12]. Treatment of *C. album* attenuated the Mercury-induced rise in oxidative stress markers, which indicates that *C. album* may have an important role in reducing Mercury toxicity. The effect of *C. album* is comparable with standard vitamin C group. Studies revealed that *C. album* contains vitamin C and E which play a vital role in hampering the absorption of Mercury in the gastrointestinal tract [11] and prevent ROS-mediated membrane damage by binding up ROS within the cell membrane [50]. It is postulated that the antioxidant CAT, SOD, and POD depletion by mercury may be a trigger for the production of reactive oxygen species (ROS) that induce lipid, protein, and DNA oxidation. As SOD, CAT and GST are the first line of defense against free radical damage [38, 51]. Decrease antioxidant content in testicular tissues made spermatogenic cells more susceptible to oxidative stress, especially during increased free radical production [52]. Hence, *C. album* by enhancing the endogenous antioxidants prevents the Mercury-induced oxidative stress.

Mercury treatment has been reported to produce a reduction in sperm quantity (testis and epididymis) and daily sperm production, followed by decrease in sperm motility while increase in head and tail morphologic abnormalities were observed [53]. Similarly, Mercury-treated group showed significantly decreased values for DSP. Previous reports indicated that vitamin C supplementation significantly increased sperm count, sperm morphology, and sperm mobility in human semen [54, 55] and/or by mitigating physiological toxicity. *C. album* extract is reported to increase sperm count and sexual behavior and also its seed extract is reported as a potent sperm immobilizing agent [20, 56].

DNA damage in sperms is assessed by comet assay. Mercury compounds have been reported to cause DNA breaks by means of free radical-mediated reactions that may lead to an increase in the occurrence of spermatozoa with anomalous heads [38]. Protection of DNA by vitamin C in sperms against endogenous oxidative damage was evident in previous reports [20]. *C. album* protects DNA damage in a manner similar to vitamin C. The antioxidant potential of *C. album* might benefit in protecting against Mercury-induced sperm DNA damage.

Regarding the hormone levels, a decrease in the plasma testosterone was observed in the Mercury-treated group. This decline in testosterone level appears to be due to a drop in the activity of enzymes engaged in the biosynthesis of testosterone or due to the diminish in testicular cholesterol, a precursor of testosterone synthesis [57]. Sperm concentration in the epididymis may decline due to miniature sperm production in the testis, which could be allied to a low level of testosterone, a chief regulator for sperm production. Similarly, growth of accessory sex glands needs testosterone [58], and the decline in the weight of these glands (Table 1) due to mercury treatment can result from the drop in the testosterone levels (Fig. 1). Histological examination has also shown a decrease in luminal spermatozoa when exposed to mercury (Figs. 2 and 3). The reduction in epididymal sperm concentration is consistent with the histologic examination of the seminiferous tubules, which showed a decrease in luminal spermatozoa in Mercury-exposed rats (Figs. 2 and 3). In the current examination, the decline in sperm number/epididymis weight and motility was allied with a rise of sperm aberrations in rats exposed to Mercury, which showed that Mercury impedes with spermatogenesis by crossing the blood-testis barrier and gaining access to germinal cells. The unfavorable effects of Mercury on mammalian testicular tissue have been unveiled with marked testicular spermatogenic degeneration at the spermatocyte level in rats [59]. Several active components in *C. album* may forage ROS generated by Mercury, lower down lipid peroxidation, and ignite the activity of antioxidant enzymes whereby leading to protection against Mercury-induced testicular damages which are manifested by an increase in sperm abnormalities and decline in testosterone level. The improvement of sperm quality may be due to the antioxidant components of *C. album*, such as α-tocopherol (vitamin E) and ascorbic acid (vitamin C) that improve testicular functions and sperm quality [60, 61].

## Conclusion

The current study results show a clear defensive mechanism of *C. album* against oxidative stress induced by Mercury and indicating the utilization of *C. album* for improving sperm production and fertility. Hence, consumption of *C. album* in diet can be a good source of functional nutrients that protect the body against heavy metal-induced oxidative stress and reproductive toxicity. Increased awareness in the society through scientific shreds of evidence may consequently increase the consumption of this nutrient-rich plant to protect against heavy metal-induced toxicities.

## Limitations of the study

Before making a certain proclamation about the suitability of *C. album* as a protective agent against Mercury-induced testicular toxicity, further studies are essential to be undertaken in order to clarify the mechanism at the molecular level. For this, western blot analysis and immunohistochemistry would provide evidence regarding signaling pathway in the pathogenesis of testicular damage and protection afforded by the plant extract. Additionally, the effect of plant extract at different doses will provide understanding on the more appropriate prescribed amount because for natural plant investigations, the dose used, especially content of antioxidants, is always blurry, not to indicate the shift dose for humans. In animal study, antioxidants are given to animals via oral or intraperitoneal injection. Bearing in mind, ROS and oxidative stress act positively in certain circumstances and the variance between animals and humans, the effective dose and safe dose, duration of treatment, absorption, and bio-availability of antioxidants require thorough investigations. Therefore, in the future, large-scale samples and appropriate duration studies against various organ toxicities and protective influence of C*. album* should be performed.

## Data Availability

The datasets used and/or analyzed during the current study are available from the corresponding author on reasonable request.

## Abbreviations

T: Testosterone FSH Follicle-stimulating hormone
E_2_: Estradiol
ROS: Reactive oxygen species
GnRH: Gonadotropin-releasing hormone
17-β-E2: 17-β-Estradiol
ER: Estrogen receptor
ELISA: Enzyme linked immunosorbent assay
BMI: Body mass index
HRP: Horseradish peroxidase
TMB: Tetra methyl benzidine
DPX: Di-*n*-butyl phthalate in xylene
POD: Peroxidase
GSTs: Glutathione-S-transferases
